# Emodin Promoted Intestinal Secretion of GLP-1 and Limited Cognitive Deficits in Young Bilateral Ovariectomized Rats

**DOI:** 10.3390/ijms27083414

**Published:** 2026-04-10

**Authors:** Xin-Yuan Liu, Chao-Yuan Ye, Yuan-Cheng Liu, Meng-Ying Zhao, Ya-Nan Li, Li Lin, Yan-Jun Du, Ying-Yan Fang, Qing Tian

**Affiliations:** 1 Key Laboratory of Neurological Disease of Hubei Province and National Education Ministry, Department of Pathology and Pathophysiology, School of Basic Medicine, Tongji Medical College, Huazhong University of Science and Technology, Wuhan 430030, China; 2 National Famous Traditional Chinese Medicine Professor Sun Guojie Inheritance Studio, Hubei Shizhen Laboratory, Hubei University of Chinese Medicine, Wuhan 430065, China; 3 Key Laboratory of Renal Diseases Occurrence and Intervention of Hubei Province, Medical College, Hubei Polytechnic University, Huangshi 435003, China

**Keywords:** emodin, GLP-1, PCSK1, proGCG, estrogen receptor β

## Abstract

Estrogen deficiency is an established risk factor for menopausal brain dysfunctions in women. Urgent exploration of drugs is needed to improve estrogen deficiency-related brain dysfunctions without the side effects of estrogen supplements. Three-month-old rats had bilateral ovariectomy (OVX) performed and were treated with emodin (EMO, 80 mg/kg/day) and 17 β-estradiol (EST, 0.5 mg/kg/day). Brain functions were evaluated by cognition and emotion-related behavioral tests. Levels of glucagon-like peptide-1 (GLP-1) and estrogen in blood, mRNA levels of estrogen receptor (ER) α, ERβ, GLP-1 receptor (GLP-1R), proprotein convertase subtilisin/kexin type 1 (PCSK1) and proglucagon (proGCG) in intestinal segments, and brain ERα and GLP-1R levels were evaluated. Contractions of isolated intestinal segments were recorded. Additionally, an ERβ antagonist, PHTPP (200 μg/kg/day), was used to clarify the role of ERβ. EST and EMO significantly ameliorated cognition deficit and depressive behaviors in OVX rats, and reduced neuronal loss and synaptic abnormalities in the hippocampus and prefrontal cortex. The blood GLP-1 levels of sham operation rats (sham, 3.09 pg/mL), EMO-treated (2.57 pg/mL) and EST-treated OVX rats (2.64 pg/mL), were higher than that of OVX rats (1.03 pg/mL). EMO had no effect on the blood estrogen level. Furthermore, EMO up-regulated mRNA levels of ERβ in ileum, colon, and cerebral GLP-1R level, while EST increased mRNA levels of ERβ in colon and cerebral ERα level. In vitro intestinal segment spontaneous contraction tests revealed that EMO reduced contraction amplitudes in isolated intestinal segments from OVX rats, with the ileum and proximal colon showing greater sensitivity to EMO. The ileum and colon segments from OVX rats were less sensitive to EST as compared to those of normal rats. Upon PHTPP intervention, the up-regulated intestinal mRNA levels of ERβ, PCSK1, proGCG, blood GLP-1 level by EMO, and the beneficial effects of EMO in abnormal behaviors of OVX rats were significantly inhibited. Overall, it was found that EMO up-regulated blood GLP-1 level via intestinal Erβ-dependent mechanism and increased brain GLP-1R level, which may be involved in the neuroprotection of EMO in OVX animals.

## 1. Introduction

Besides the important roles in female reproduction, estrogen is also involved in regulating non-reproductive systems such as the nervous system and body weight homeostasis. In the brain, estrogen participates in neuronal survival and synaptic plasticity required for cognition and emotion via estrogen receptors (ERs), mainly ERα and ERβ [1]. Estrogen deficiency increases the risk for dementia and depression in both women and female rodents [2,3]. Many studies have confirmed that estrogen prevents dementia and depression and regulates body weight homeostasis [4,5], although data from some clinical trials, including the Women’s Health Initiative Memory Study, Kronos Early Estrogen Prevention Study, Women’s Health Initiative Hormone Therapy trial, and the Early vs. Late Intervention Trial of Estradiol, were not encouraging. However, estrogen supplementation in postmenopausal women was reported to increase the occurrence of breast cancer, cardiovascular diseases, and cognitive impairment [6]. As such, safer and more effective therapies for clinical management of brain dysfunction in the setting of estrogen deficiency warrant urgent development.

As a natural anthraquinone derivative, emodin (EMO) is present in the roots of *Polygonum cuspidatum* and *Rheum palmatum*, as well as the *Polygonum multiflorum* vine and the leaf of *Aloe vera*; these are among the most widely used herbs in traditional Chinese medicine [7,8]. Multiple biological activities of EMO have been reported, including inhibition of apoptosis, induction of autophagy, decreasing β-amyloid (Aβ) levels, and reducing tau overload and neuroinflammation, thus suggesting significant neuroprotective potential [9,10]. EMO improved depressive behaviors of animals subjected to chronic, irregular mild stress, likely via up-regulating levels of glucocorticoid receptor and brain-derived neurotrophic factor, inhibiting neuroinflammation and regulating phosphatidylinositol-3-kinase (PI3K)/protein kinase B (PKB) interactions [11,12]. These findings suggest that EMO may play a protective role in estrogen deficiency-related brain dysfunctions. In a study of Aβ-induced neurotoxicity, the neuroprotective effects of EMO were blocked by pre-treatment with an ER antagonist ICI182780 [13]. By docking studies, EMO was suggested to bind to ERβ with a higher binding affinity score [14]. In ER*α* activation-associated breast cancer, EMO inhibited the proliferation of cancer cells by down-regulating ER α protein levels, thereby suppressing ERα transcriptional activation [15]. These reports indicated the mechanistic connections between EMO effects and ERβ.

Glucagon-like peptide-1 (GLP-1), an incretin hormone, has demonstrated neuroprotective consequences by activating the GLP-1 receptor (GLP-1R) in the brain [16,17,18]. The intestinal enteroendocrine L cells are the primary source of GLP-1, a peptide hormone produced by proprotein convertase subtilisin/kexin type 1 (PCSK1) cleaving proglucagon (proGCG) [19]. The function of L cells was indicated as ER related [20]. Bilateral ovariectomy (OVX) mice were reported to have decreased GLP-1 secretion and level, which was reversed by estradiol treatment. An increase in estradiol GLP-1 secretion from mouse and human intestinal explants occurred only by ERβ activation [21]. GLP-1 ameliorates sucrose-induced obesity and glucose intolerance in OVX mice [22]. *Rheum* and *Polygonum cuspidatum* are commonly used herbs in treating intestinal dysfunctions, and EMO is the most extensively studied monomer [8,23]. Based on the above studies, we presumed that EMO might improve OVX-induced brain dysfunctions, partially by promoting intestinal GLP-1 secretion in an ERβ-related manner.

In this study, OVX was performed to construct a rat model of estrogen deficiency. Effects of EMO and 17β-estradiol (EST) on the behaviors of OVX rats were subsequently observed. EMO (6 weeks; 80 mg/kg/day) and EST (6 weeks; 0.5 mg/kg/day) gavage both rescued cognitive deficits and depression-like behaviors of OVX rats while alleviating the neuronal loss and synaptic deficits in the hippocampus and prefrontal cortex (PFC). While EMO administration increased GLP-1 content in peripheral blood and the levels of GLP-1R in brain of OVX rats, EST administration additionally increased levels of circulating estrogen and cerebral ERα. Under physiological conditions, L cells appear more frequently in the epithelial layer of the ileum and colon. In vitro spontaneous contraction tests of intestinal anatomical segments revealed that EMO dose dependently reduced contraction amplitudes in this rat population, with the ileum and proximal colon most sensitive to the effects of this compound. However, in OVX rats, the duodenum, ileum, and proximal colon were less sensitive to EST as compared to control rats. Since EMO’s mechanism of action did not involve ERα, we further employed an ERβ antagonist PHTPP (4-[2-phenyl-5,7-bis (trifluoromethyl) pyrazolo [1,5-a]pyrimidin-3-yl]phenol, 6 weeks; 200 μg/kg/day) to pretreat OVX rats receiving EMO treatment, aiming to verify whether EMO improves brain function in OVX rats via an ERβ dependent way. Following the blockade of ERβ, the effects of EMO in promoting intestinal GLP-1 secretion and improving brain dysfunctions were eliminated. As such, EMO promoted Erβ-dependent intestinal GLP-1 production and increased brain GLP-1R level. The enhanced GLP-1/GLP-1R signaling may be one of the important pathways for EMO to improve the brain functions in estrogen-deficient rats.

## 2. Results

### 2.1. EMO Alleviated the Spatial Cognitive Deficits of OVX Rats

**Figure 1:**
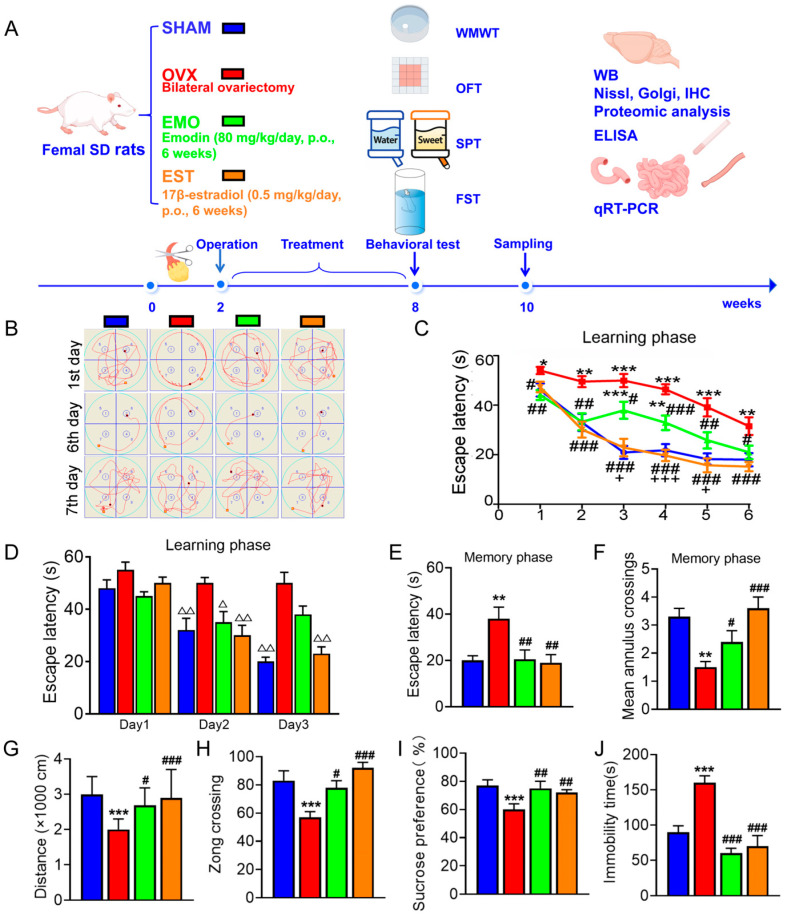
EMO prevents cognitive deficit and depressive behaviors in OVX rats. SD (3-month-old, female) rats were divided into sham (n = 16, sham) and ovariectomized (n = 48, OVX) groups. Two weeks after surgery, OVX rats were treated with either EMO (n = 16, 80 mg/kg/day, p. o.) or EST (n = 16, 0.5 mg/kg/day, p. o.) for six consecutive weeks. (**A**) Schematic diagram. (**B**) Swimming paths and (**C**,**D**) latencies for platform searching during the learning phase of Morris Water Maze test (MWMT). (**E**) Latency of first-time platform crossing and (**F**) average annulus crossings within 1 min during the memory phase of MWMT. (**G**) Total distance traversed and (**H**) number of zone crossing in open-field test (OFT). (**I**) Sucrose consumption percentage in sucrose preference test (SPT). (**J**) Immobility time in forced swimming test (FST). Data are presented as mean ± SEM. * *p* < 0.05, ** *p* < 0.01, *** *p* < 0.001, vs. sham rats; # *p* < 0.05, ## *p* < 0.01, ### *p* < 0.001, vs. OVX rats; + *p* < 0.05, +++ *p* < 0.001, vs. EMO rats. Δ *p* < 0.05, ΔΔ *p* < 0.01, vs. the records of the previous day in the same group.

In the preliminary works, we observed the effects of different concentrations of EMO (20 mg/kg/day, 40 mg/kg/day, 80 mg/kg/day) on the cognitive impairment in OVX rats. Six weeks of EMO treatment (80 mg/kg/day) showed the best brain protections in OVX rats (Supplementary Figure S1). Here, we compared the effects of EMO (80 mg/kg/day) and EST (EST, 0.5 mg/kg/day) on OVX rats after 6 weeks’ treatments (Figure 1A).

During the spatial learning phase of Morris Water Maze test (MWMT), OVX rats spent more time reaching the platform as compared to sham, EMO, and EST rats (Figure 1B,C). Compared with the previous day’s records, EMO rats showed no difference in learning on the second day compared to EST and sham rats, but their learning performance on the third day was worse than that of EST and sham rats (Figure 1D). Both EST and sham rats learned to quickly reach the platform on the third day. EMO rats did not perform as well as EST rats on third, fourth, and fifth days, but reached the level of EST rats on sixth day. During the memory testing phase, OVX rats spent much longer reaching the target region and passed through the target region less frequently. No differences were observed between sham, EMO, and EST groups (Figure 1E,F). In open-field test (OFT) (Figure 1F,G), no significant differences between sham, EMO, and EST rats were noted, although OVX rats moved shorter distances and traversed fewer squares. While OVX rats displayed no obvious preference for sugar water in sucrose preference test (SPT) (Figure 1G) and had prolonged immobility in forced swimming test (FST) (Figure 1H), no differences in sugar water preference and immobility time were noted among sham, EMO, and EST rats (Figure 1I,J).

**Figure 2:**
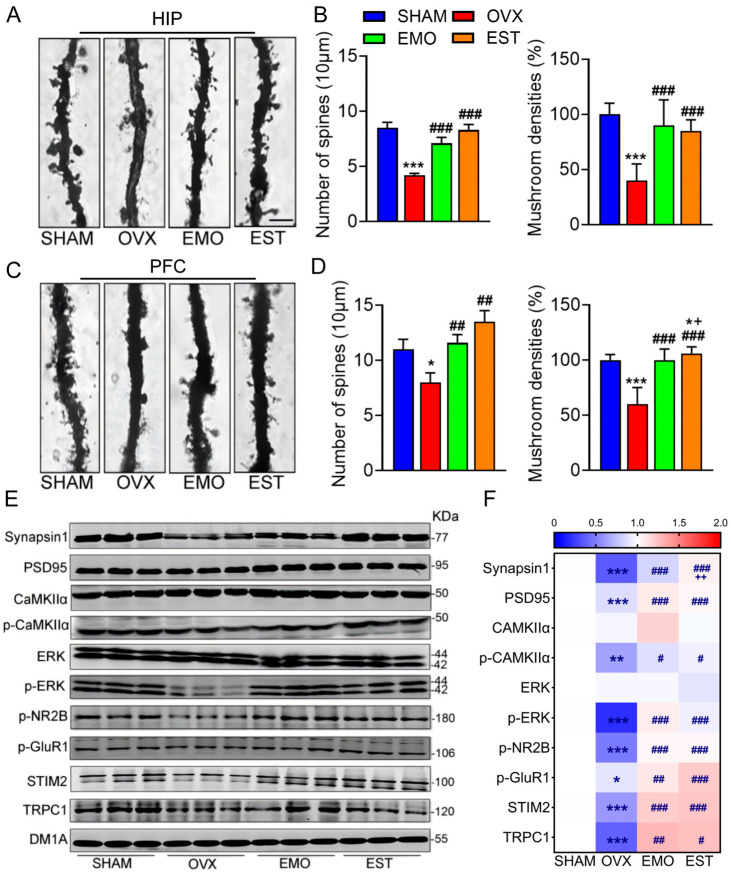
EMO attenuates synaptic deficits in OVX rats. Eight weeks after surgery, neuronal spines in hippocampus ((**A**), scale bar = 5 μm) and PFC ((**C**), scale bar = 5 μm) were detected via Golgi staining. Quantitative analysis of spine numbers and mushroom-shape densities in hippocampus (**B**), PFC (**D**,**E**), Western blotting, and (**F**) quantitative analysis of Synapsin 1, PSD95, CaMKIIα, p-CaMKIIα, ERK, p-ERK, p-NR2B, p-GluR1, STIM2, and TRPC1 in the hippocampus. Normalization was performed using the relative level of the sham group as the reference (n = 6/group). Data are presented as mean ± SEM. * *p* < 0.05, ** *p* < 0.01, *** *p* < 0.001, vs. sham rats; # *p* < 0.05, ## *p* < 0.01, ### *p* < 0.001, vs. OVX rats; + *p* < 0.05, ++ *p* < 0.01, vs. EMO rats.

Using Nissl staining, the loss of neurons was observed in hippocampal CA1 and CA3 regions and PFC of OVX group rats (Supplementary Figure S2). Using Golgi staining, the density of spines on neurites was shown to be decreased by 50.06% in the hippocampus (Figure 2A,B) and 25.76% in PFC among OVX group rats (Figure 2C,D), compared to the sham group, while the number of mushroom-type spine, linked to cognition, decreased by 56% in the hippocampus (Figure 2B) and 37% in PFC (Figure 2D). Furthermore, the significant reductions in presynaptic (synapsin 1) and postsynaptic (PSD95) molecules in the hippocampus of OVX rats were noted (Figure 2E,F). In contrast, spine density and mushroom-shape proportion in brains of EMO and EST rats revealed no significant differences as compared to the sham rats (Figure 2E,F).

The CaMKII (Ca^2+^/calmodulin-dependent protein kinase II) cascade and ERK (extracellular signal-regulated kinase) cascade are important pathways underlying synaptic plasticity, neuronal excitability, learning, and memory. CaMKII is the most abundant protein in excitatory synapses and considered a central molecular organizer of synaptic plasticity, learning, and memory [24,25]. The CaMKII-NR2B (2B subunit of NMDA (N-methyl-D-aspartate receptor)) complex initiates a structural rearrangement of the postsynaptic density (PSD, a membrane thickening juxtaposed to the presynaptic active zone) by a mechanism requiring PSD-95 and GluR1 (Glutamate receptor 1) [26]. GluR1, an AMPA glutamate receptor subunit (also known as GluA1), is required for synaptic plasticity and spatial memory retention [27,28]. CaMKII, NR2B, and GluR1 have been found to underlie a range of neurological disorders, and CaMKII colocalizes with the endoplasmic reticulum Ca^2+^ sensor protein, STIM2 (Stromal interaction molecule 2) [29]. ERK signaling is reported to be critical in estrogen-associated neuronal survival and synaptic plasticity [30,31]. The cytosolic free Ca^2+^ plays a pivotal role in regulation of neuronal excitability, synaptic plasticity, and neurotransmitter release, and its levels are regulated not only by the channels located in the plasma membrane but also by the endoplasmic reticulum. TRPC1 (transient receptor potential channel 1) and STIM2 are two important Ca^2+^ channels on endoplasmic reticulum membrane and closely related to neuronal excitability [32,33]. Levels of hippocampal TRPC1 and STIM2 and activation of CaMKIIα and ERK cascades were shown to be decreased in OVX rats, and the significant reductions in phosphorylated NR2B and GluR1 were also observed. Reductions in the aforementioned proteins, however, were not detected in the hippocampi of the EMO and EST group rats (Figure 2E,F).

Taken together, these findings indicate that both EMO and EST reduced the synaptic damages and ameliorated spatial cognitive deficits and depression-like behaviors in OVX rats.

### 2.2. EMO Increased Levels of Blood GLP-1 and Brain GLP-1R in OVX Rats

**Figure 3:**
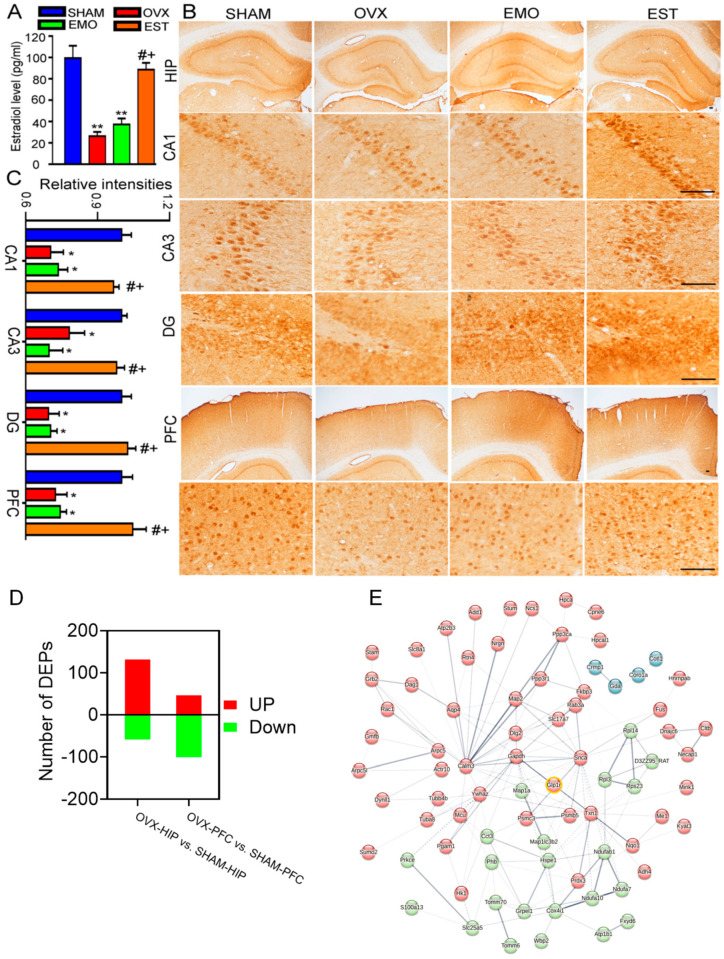
EMO exerts no effects on decreased levels of blood 17β-estradiol and cerebral ERα in OVX rats. After the MWMT, plasma 17β-estradiol was detected using ELISA (**A**), and brain slices were prepared for ERα immunohistochemical detection ((**B**), scale bar = 200 μm) and quantitatively analyzed (n = 3/group, (**C**)). (**D**) Number of up-regulated and down-regulated differentially expressed proteins (DEPs) in the hippocampus and PFC of OVX rats compared with sham rats (n = 6/group). (**E**) Protein–protein interaction (PPI) network of DEPs associated with GLP-1R. Data are presented as mean ± SEM. * *p* < 0.05, ** *p* < 0.01, vs. sham rats; # *p* < 0.05, vs. OVX rats; + *p* < 0.05, vs. EMO rats.

As estrogen is beneficial for the brain, we then tested the blood estrogen levels of rats. The average estrogen level was 26.26 pg/mL in OVX rats and 37.30 pg/mL in EMO rats, significantly less than those in the sham (99.31 pg/mL) and EST (88.53 pg/mL) rats (Figure 3A). While OVX rats had decreased hippocampal ERα levels, this was not the case for ERβ [34]. In the hippocampus (CA1, CA3, and DG) and PFC, ERα intensities in OVX and EMO rats were shown to be less than those in the sham and EST rats (Figure 3B,C). These findings indicate that the brain function improvement of EMO is not related to estrogen signals in the brain.

**Figure 4:**
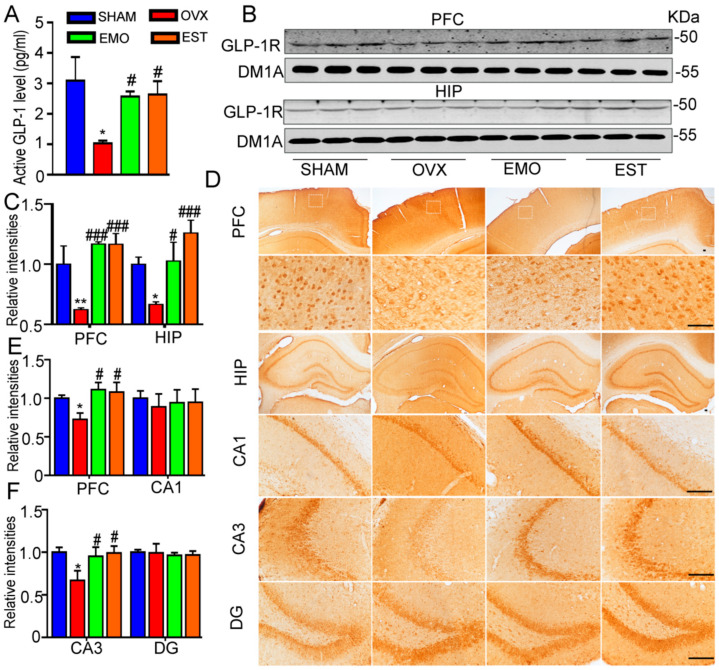
The blood active GLP-1 levels and cerebral GLP-1R levels in rats from sham, OVX, EMO, and EST groups. After behavioral tests, (**A**) the GLP-1 level was detected (n = 8/group). (**B**) Western blotting and (**C**) quantitative analysis of GLP-1R in the hippocampus and PFC (n = 6/group), (**D**) GLP-1R immunohistochemical staining in brain slices, and (**E**,**F**) quantitative analysis (n = 6/group; scale bar = 200 μm) were performed. Data are presented as mean ± SEM. * *p* < 0.05, ** *p* < 0.01, vs. sham rats; # *p* < 0.05, ### *p* < 0.001, vs. OVX rats.

To explore this potential mechanism, we performed a proteomic analysis of brain samples from OVX rats and sham rats. There were 3208 proteins quantitatively analyzed in total. Using an iTRAQ ratio of >1.15, coupled with *p* < 0.05 as the up-regulated threshold and <0.87 coupled with *p* < 0.05 as the down-regulated threshold, 362 differentially expressed proteins (DEPs) were obtained in the OVX rats, namely, 52 up-regulated and 107 down-regulated DEPs in PFC, and 138 proteins up-regulated and 65 proteins down-regulated DEPs in the hippocampus (Figure 3D). Using PPI analysis, the GLP-1R signal was suggested and associated with several DEPs in the OVX brain (Figure 3E), which prompted us to further assess circulating GLP-1 and cerebral GLP-1R levels. The blood levels of active GLP-1 in OVX rats (1.03 pg/mL) were significantly decreased. GLP-1 levels in EMO (2.57 pg/mL) and EST (2.64 pg/mL) rats did not differ significantly from those of the sham (3.09 pg/mL) rats (Figure 4A). Western blotting revealed the lower levels of GLP-1R in the hippocampus and PFC of OVX rats as compared to sham (Figure 4B,C), which was further confirmed by the immunohistochemical testing on brain slices. Importantly, cerebral GLP-1R levels in EMO rats did not significantly differ from the sham and EST rats (Figure 4D–F). These data suggest that EMO increased the blood GLP-1 level and cerebral GLP-1R level, without affecting circulating estrogen or cerebral ERα.

### 2.3. EMO Up-Regulated mRNA Levels of proGCG and PCSK1 in Ileum and Colon and Induced Greater Changes in Intestinal CAs of OVX Rats

**Figure 5:**
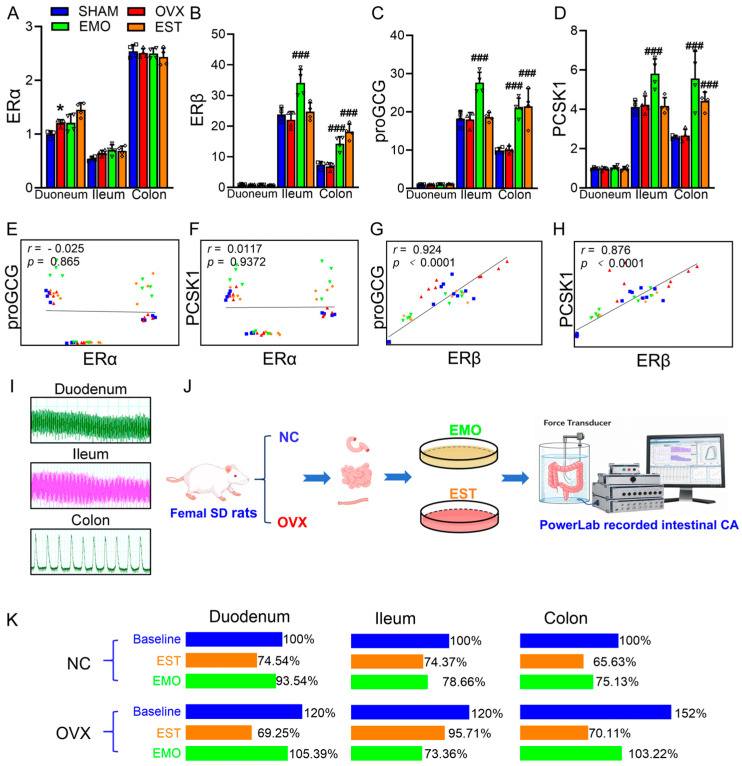
Effects of EMO and EST on intestinal mRNA levels of ERα, ERβ, proGCG, and PCSK1 and intestinal spontaneous contractions. (**A**–**D**) mRNA levels of ERα, ERβ, proGCG, and PCSK1 in the duodenum, ileum, and colon of rats (n = 6/group). The ordinate represents fold change, normalized to the level of the duodenum in sham group as 1.0. * *p* < 0.05, vs. sham rats; ### *p* < 0.001, vs. OVX rats. (**E**–**H**) Pearson correlation scatter plots of the associations between intestinal ER mRNA levels with proGCG mRNA and PCSK1 mRNA levels. Correlation coefficients (*r*) and *p* values are indicated in each plot. The individual data points are shown as open symbols in panels (**A**–**D**), and as solid symbols in panels (**E**–**H**). (**I**) Representative tension waves of spontaneous contractions in isolated duodenum, ileum, and colon segments. (**J**) Schematic diagram. (**K**) Changes in intestinal contraction amplitudes (CA) in NC and OVX rats after EST or EMO treatment (n = 6/group). The baseline CA of NC were set as 100%. Data are presented as mean ± SEM.

Both EMO and estrogen treatments up-regulated blood GLP-1 levels. Estrogen was reported to promote L cell secretion of GLP-1 via ERβ [21]. To explore the EMO-related mechanism, we measured mRNA levels of ERα (Figure 5A), ERβ (Figure 5B), proGCG (Figure 5C), and PCSK1 (Figure 5D) in the duodenum, ileum, and colon. Only the increased ERα mRNA levels in the duodenum was observed in OVX rats. When compared to OVX rats, the higher mRNA levels of ERβ, proGCG, and PCSK1 in the ileum and colon were detected in EMO rats, while the increased ERα mRNA level in ileum and increased ERβ, proGCG, and PCSK1 mRNA levels in colon were shown in EST rats. These data indicated that EST and EMO up-regulate the level of GLP-1 through different ways. Then, we analyzed the correlations between mRNA levels of ER subtypes with the mRNA levels of proGCG and PCSK1 in intestinal samples (Figure 5E–H). It was shown that proGCG mRNA level (Figure 5G) and PCSK1 mRNA level (Figure 5H) were positively correlated with ERβ mRNA level.

A previous study reported the unaffected intestinal transit in OVX rats [35]. Here, the intestinal motility, e.g., contractive frequency (CF) and contractive amplitude (CA), of duodenum, ileum, and colon were evaluated. In the NC group, the CFs were 35.3, 26.49, and 2.33 times/min for duodenum, ileum, and colon respectively, with no significant differences between NC and OVX groups. Neither EST nor EMO affected CFs in any segment. Then we treated the segments with EST or EMO and recorded the change rates of CA (Figure 5I,J). Both EST and EMO showed CA inhibition in the isolated intestinal segments. As shown, in both NC and OVX groups, EST treatment induced greater changes in CA of the duodenum and colon than EMO. However, in the ileum of the OVX group EMO induced a greater change in CA than EST, although EST treatment showed no difference with EMO in ileum CA of NC. In OVX intestinal segments, EMO decreased 38.9% in CA of ileum and 32.1% in CA of colon (Figure 5K).

### 2.4. PHTPP Suppressed EMO-Induced Improvements in OVX Rats

**Figure 6:**
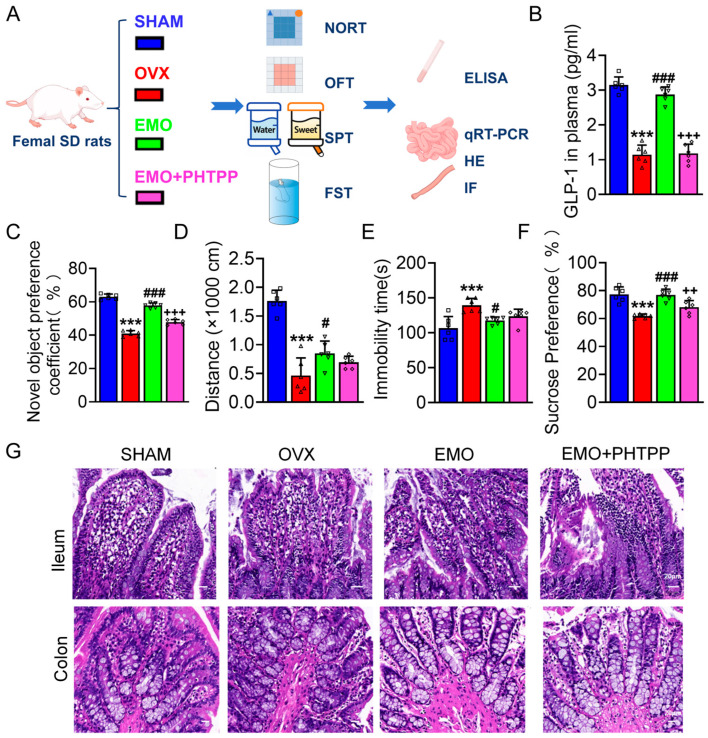
PHTPP attenuated the beneficial effects of EMO in OVX rats. (**A**) Schematic diagram. (**B**) Blood active levels of GLP-1. (**C**) Object preference coefficient in novel object recognition test (NORT). (**D**) Total distance traveled in OFT. (**E**) Immobility time in FST. (**F**) Sucrose consumption percentage in SPT. (**G**) Representative H&E staining of ileal and colonic tissues (scale bars: 200 μm for overview, 50 μm for insets). The individual data points are shown as open symbols in panels (**B**–**F**). Data are presented as mean ± SD, *** *p* < 0.001, vs. sham rats; # *p* < 0.05, ### *p* < 0.001, vs. OVX rats; ++ *p* < 0.01, +++ *p* < 0.001, vs. EMO rats (n = 6/group).

**Figure 7:**
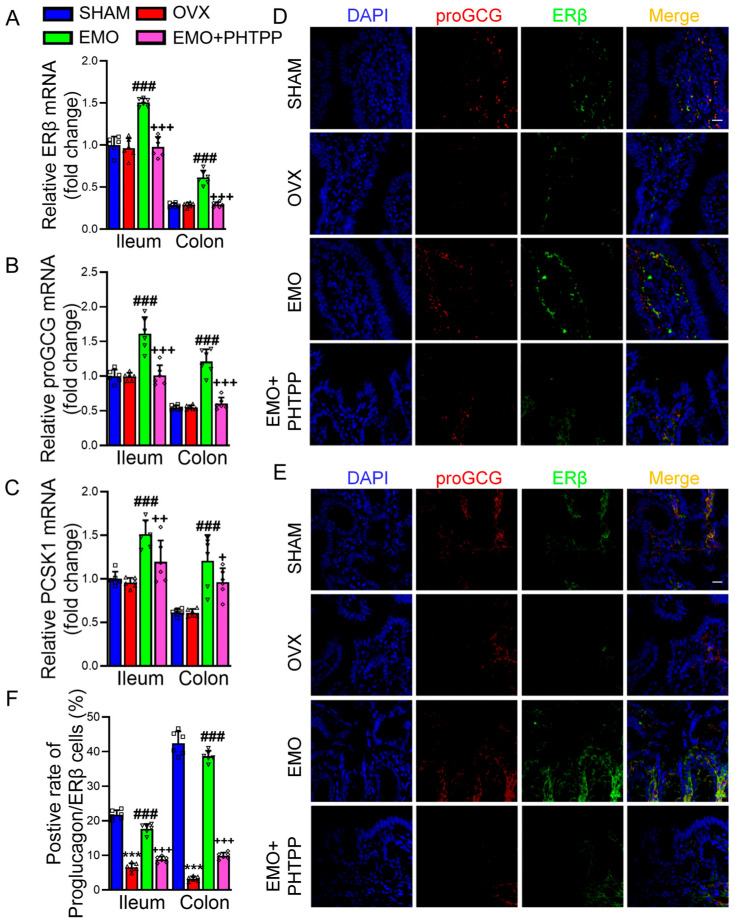
EMO promoted ERβ-proGCG co-localization in intestinal epithelial cells, an effect attenuated by PHTPP. The mRNA levels of ERβ (**A**), proGCG (**B**), and PCSK1 (**C**) in the ileum and colon of rats from sham, OVX, EMO, and EMO + PHTPP groups. (**D**,**E**) Immunofluorescence staining of proGCG (red) and ERβ (green), and DAPI staining (blue) in ileum and colon (scale bar = 20 μm). (**F**) Quantitative analysis of proGCG/ERβ double-positive cell rate in ileum and colon (n = 6/group). Data are presented as mean ± SD, *** *p* < 0.001, vs. sham rats; ### *p* < 0.001, vs. OVX rats; + *p* < 0.05, ++ *p* < 0.01, +++ *p* < 0.001, vs. EMO rats (n = 6/group).

To examine the involvement of ERβ in the increase in GLP-1 by EMO in OVX rats, the selective ERβ antagonist PHTPP was used as shown in Figure 6A. PHTPP attenuated the increase in GLP-1 by EMO in OVX rats (Figure 6B). No improvements of behavioral improvements were observed in EMO plus PHTPP-treated OVX rats (EMO + PHTPP, Figure 6C–F). HE staining (Figure 6G) of the ileum and colon showed OVX rats exhibited disrupted mucosal structure, shortened villi (ileum), and disorganized glandular architecture (colon), whereas EMO reduced the abnormal morphological changes. In contrast, the alleviation was much less obvious in EMO + PHTPP rats. In addition, the mRNA level analysis revealed that the up-regulations of ERβ, proGCG, and PCSK1 levels in the ileum and colon by EMO were inhibited in EMO+PHTPP rats (Figure 7A–C).

Immunofluorescence staining revealed distinct co-localization (yellow) patterns of ERβ (green) and proGCG (red) in the ileum (Figure 7D) and colon (Figure 7E). In the sham rats, intense yellow co-localization signals were observed in crypt epithelial cells, with proGCG/ERβ double-positive cells accounting for 21.3% (ileum) and 44.6% (colon) of total epithelial cells. OVX significantly reduced levels of proGCG and ERβ and their co-localization (Figure 7F). EMO treatment increased the levels of proGCG and ERβ and their co-localization in the ileum and colon of OVX rats, which were completely abolished by PHTPP. These results support that the up-regulation of GLP-1 by EMO is intestinal ERβ-dependent.

## 3. Discussion

Estrogen deficiency induces brain dysfunction, especially in females. Previously, we reported decreased levels of circulating estrogen and hippocampal ERα in OVX rats [34]. Estrogen receptor agonists are known to alleviate neuroinflammation and neurodegeneration. However, side effects limit clinical applicability of estrogen replacement. Similarly, GLP-1, a gut-derived peptide hormone, significantly decreased after menopause [36,37], possesses neuroprotective properties [38]. In this study, EMO and EST were found to increase the diminished levels of blood GLP-1 and cerebral GLP-1R in OVX rats, which aligns with previous evidence that EMO upregulates GLP-1R expression in vivo [39].

Our findings reveal that EMO mimics the effects of EST by up-regulating the mRNA levels of GLP-1 production related molecules, proGCG and PCSK1, through ERβ in intestinal L cells, particularly in the ileum and colon where L cell density is highest. This is consistent with previous reports that EST promotes GLP-1 secretion via ERβ [21]. However, unlike EST, EMO achieves this without increasing estrogen levels, suggesting its unique advantage as a non-hormonal alternative. Furthermore, our observation that EMO reduces intestinal CA in OVX rats provides a plausible explanation for its ability to enhance GLP-1 secretion. By slowing intestinal motility, EMO may prolong nutrient contact with L cells, thereby facilitating GLP-1 release [40]. This reconciles seemingly contradictory reports of EMO’s effects on gut motility [41,42], emphasizing the importance of model and region-specific responses. Lack of experimental data from the cultured estrogen deficient L cells to demonstrate the effect of EMO on GLP-1 secretion is a limitation of this study.

EMO is a multi-target molecule. In addition to promoting GLP-1 synthesis, previous studies have reported that EMO inhibits the activity of dipeptidyl peptidase-4 (DPP4) [43], an enzyme that cleaves polypeptides, such as neuropeptides, peptide hormones, and chemokines, and thus is responsible for degrading active GLP-1. DPP-4 is found expressed in kidney, lung, placenta, intestines, heart, brain, liver, lung, skeletal system, endothelium, and immune system, and also exists soluble in blood. In humans, serum DPP4 levels were reported positively correlated with an increased body mass index (BMI), fat volume, and insulin concentration. The visceral fat accumulation is a notable phenotype of estrogen deficiency; DPP4 expression in the perigonadal fat was reported no differences between OVX mice with a high-fat diet and control [44]. In this research, we detected the increased active GLP-1 levels in blood of EMO rats and EST rats, which cannot rule out the possibility that EST and EMO inhibit DPP4 activity. The levels of DPP-4 activation in blood and brain of OVX animals should be further assayed. EMO is a stimulant laxative by up-regulating the PKA/p-CREB signal to increase expression of aquaporin 3 (AQP3) in the colon [45]. Here, we observed that EMO induced a greater CA change than EST in colon of OVX rats. Further studies are warranted on the intestinal contraction, L cells functions, and the intestinal environment during estrogen deficiency, which helps to deeply understand the protective mechanism of EMO in OVX rats.

GLP-1 readily crosses the blood–brain barrier (BBB) and disperses throughout the cerebral parenchyma [46]. Its receptor, GLP-1R, is known to be widely expressed in neuronal tissue [47]. Hippocampal GLP-1R expression in mice fed a high-fat diet was further enhanced by puerarin treatment, which subsequently improved neuroplasticity [48]. GLP-1 was reported to suppress apoptosis via PI3K/PKB/mTOR signaling, while the GLP-1R agonist liraglutide was reported to protect apoptosis due to high concentrations of glucose [49,50] and reduce neurodegeneration [51]. A long-acting GLP-1R agonist, dulaglutide, was reported to activate cAMP/PKA signaling, underscoring its potential clinical use in the management of depression [52]. Exenatide, another GLP-1R agonist, was reported to prevent memory deficits while alleviating anxiety and depressive behaviors, likely via increasing hippocampal CaMKII and PSD95 [53]. We found that OVX induced deficiencies in circulating GLP-1 levels and cerebral GLP-1R expression, with EMO and EST treatments having improved those deficiencies.

In our preliminary works, we observed the effects of different oral concentrations of EMO (20 mg/kg/day, 40 mg/kg/day, 80 mg/kg/day) in OVX rats and observed that 80 mg/kg/day of EMO (6 weeks) showed the best brain protections (Supplementary Figure S1); 80 mg/kg/day of EMO also showed brain protections in the dementia animals [10] and depression animals [12]. We did not observe EMO-induced hepatic, renal and gastrointestinal injuries in these studies. Although we did not study EMO pharmacokinetics in OVX rats in this research, previous pharmacokinetic analysis revealed rapid oral absorption of EMO in rats, with the initial plasma peak at 0.5 h [54]. A large proportion of orally administered EMO is distributed in the gastrointestinal tract, with the highest concentration in the colon, followed by the ileum [55]. In intestinal segments of OVX rats of this study, EMO decreased 38.9% in CA of the ileum and 32.1% in CA of the colon, faintly suggesting a selective distribution of EMO in OVX rats.

## 4. Materials and Methods

### 4.1. Animals and Treatment

Female Sprague-Dawley (SD; 3-month-old) rats were cared for in accordance with the Regulations on Laboratory Animal Management of China. All rats used in this study were obtained from the Experimental Animal Center, Huazhong University of Science and Technology (HUST), and were maintained in standardized animal rooms at constant temperature (24 ± 2 °C, with lights on daily 07:00–19:00). A total of four rats were housed in one cage. All experiments were approved by the Animal Care and Use Committee of Huazhong University of Science and Technology (No. 2015042901739).

A rat model of estrogen deficiency was constructed via bilateral OVX [34]. A total of 100 female SD rats were used, and all rats were randomly assigned to each group using a random number table method. In Experiment I, studying the effects of EMO in OVX rats (Figure 1A), 48 rats received OVX surgery, and 16 rats received a sham operation (sham group). Two weeks after surgery, the OVX rats were randomly divided into three groups: untreated group (OVX, n = 16), EMO treated group (EMO, 80 mg/kg/day, p.o., n = 16), and 17β-estradiol treated group (EST, 0.5 mg/kg/day, p.o., n = 16). In Experiment II, studying the effects of EMO in intestinal segments, 6 normal rats (NC) and 6 OVX rats were used. In Experiment III, studying the effects of PHTPP, 6 sham rats and 18 OVX rats were used. Eighteen OVX rats were randomly divided into three groups: untreated group (OVX, n = 6), EMO group (n = 6, 80 mg/kg/day), and EMO + PHTPP group (n = 6, 80 mg/kg/day EMO + 200 μg/kg/day PHTPP).

### 4.2. Drugs and Primary Antibodies

The EST was obtained from Sigma Aldrich (E8875, purity ≥ 98%; Merck KGaA, Darmstadt, Germany). EMO (C_15_H_10_O_5_, MW: 270.23, HPLCZ: 98%) was obtained from Yiji Co. (Shanghai, China). PHTPP (CAS 805239-56-9, purity > 99%; MCE, Monmouth Junction, NJ, USA). The information of the primary antibodies is shown in Table S1.

### 4.3. Behavior Assessment of Rats

As previously described [34], the emotional behaviors of rats were assessed via OFT, SPT, and FST; the spatial cognitive behaviors were assessed via MWMT, and the object recognition cognitive behaviors were assessed via novel object recognition test (NORT).

To assess the locomotor activity and anxiety-like behaviors, OFT was performed in a black-painted square box (100 cm in length, 100 cm in width, 40 cm in height), with the floor divided into 25 equal squares via a video tracking system. Rats were acclimated to the test room for 1 h before testing. The box was cleaned with 75% ethanol between trials. Each rat was placed in the center of the box and the behaviors were recorded during the following 5 min, including total moving distance, moving duration, zone crossings, and moving speed.

SPT was used to evaluate the depressive behaviors. Rats were acclimated to a two-bottle housing setup, then trained to drink 1.5% sucrose solution for 1 h daily. After 24 h of water deprivation, rats underwent a 1 h test with pre-weighed equal volumes of 1.5% sucrose solution and tap water. Sucrose preference was calculated as the percentage of sucrose intake relative to total liquid intake.

FST was used to assess depressive despair behavior. Rats were individually placed in a transparent Pyrex tank (40 cm in height, 30 cm in diameter) with 25 ± 2 °C water for a 5 min test. Water was replaced between trials. Immobility and moving time were recorded via video tracking.

MWMT includes a 6-day learning test and a 1-day memory test. During the learning phase, the trails of rats in a pool (25 °C water with black non-toxic ink) with 1.8 m in diameter were recorded. During the memory phase, the platform was removed for a 1 min probe test. Escape latency, swimming trails, latency to the original platform area, platform crossings, and swimming speeds were recorded.

In NORT, rats were acclimated to the test room for 1 h before testing. During the training phase, two identical objects were placed symmetrically in the box, and each rat was allowed to explore for 5 min. During the test phase, one familiar object was replaced with a novel object, and the rat was allowed to explore for 5 min. During the time spent exploring the novel and familiar objects, a discrimination index was recorded and analyzed.

### 4.4. Brain Tissue Protein Extraction and Western Blotting

Brain tissues (hippocampus and PFC) were removed from rats deeply anesthetized with isoflurane [34]. Protein concentrations in tissue samples were assessed via the bicinchoninic acid method. Proteins were separated into different bands via 10% SDS-PAGE electrophoresis, and the bands were transferred onto nitrocellulose membranes for primary antibody probing. Membranes were rinsed with Tween-20/Tris-buffered saline (Beyotime Biotechnology Co.; Shanghai, China) buffer three times and incubated with secondary antibodies (i.e., anti-R or anti-M, dilution ratio 1:10,000, conjugated to IRDye 800CW, Li-Cor Bioscience, Lincoln, NE, USA) for 1 h. Finally, rinsed membranes were visualized and quantitatively evaluated using an imaging and quantification system (Li-Cor Bioscience, USA). The densities of protein bands were normalized to the densities of the corresponding α-tubulin (DM1A) bands. The relative level of each band was calculated relative to the mean level of sham group, which was set as 1.0.

### 4.5. Brain and Intestinal Slices Preparation and Staining

Rats were deeply anesthetized with isoflurane and perfused intracardially with normal saline (100 mL) and paraformaldehyde (4%, 400 mL). Cerebral tissue and intestinal tissue were carefully removed and placed into 4% paraformaldehyde (4 °C) for further fixation. After dehydration with sucrose, brain tissue was coronally sliced into 25 µm thick slices, and intestinal tissue including those of duodenum, ileum, and colon were transversely sliced into 7 µm thick slices, which were then collected and stored at −20 °C in 50% glycerin solution). Nissl, Golgi, HE, immunohistochemistry, and immunofluorescence staining were subsequently performed. All images were obtained via microscopy with slice-scanning (Olympus VS200, Tokyo, Japan) and quantitatively analyzed using Image-Pro Plus 6.0 (Media Cybernetics; Rockville, MD, USA). For image analysis, 5 randomly selected fields per slice (3 slices/rat, 3 rats/group) were taken. The relative optical intensities, double-positive cells rate, and spine densities were quantified using a unified threshold setting for all slices. All image quantifications were performed by two independent, blinded researchers.

### 4.6. Circulating GLP-1 and Estrogen Assays

An active GLP-1 assay kit (v2) and an electrochemical luminescence analyzer (Meso Scale Discovery; Olathe, KS, USA) were employed to determine blood GLP-1 levels. Blood EST levels were determined using an ELISA kit (Elabscience Biotechnology Co.; Wuhan, China).

### 4.7. Intestinal Segment Culture and Assessment of Spontaneous Contractility

After deep anesthesia, the rats underwent a sagittal incision through the midline of the abdomen. The duodenum, ileum, and colon were quickly removed and placed into dishes containing Kreb’s solution. Intestinal anatomical segments were prepared, ensuring that each segment was approximately 1.5 cm in length. The intestinal anatomical segments were vertically immersed into automatic organ baths (LSI Letica Scientific Instruments, Barcelona, Spain) at 37 ± 0.5 °C in chambers containing Kreb’s solution (5 mL) under 5% CO_2_ and 95% O_2_ conditions.

Force transducers were used to measure isometric contractions. Spontaneous contraction curves, frequencies, and amplitudes were recorded by a specialized data acquisition and analysis system (PowerLab, Bella Vista, Australia). After 20 min of stabilization at a resting tension (1.0 g), compound administration into the organ chamber was accomplished using a microsample needle. Contractions were recorded 5 min before and after each administration. After experimentation, intestinal anatomical segments were dried with filter paper and weighed. To observe direct effects on basal intestinal contractility, EST (1.00, 5.00 and 10.00 μmol/L; 1 μL) and EMO (1, 5, 10, 20 and 50 μmol/L; 1 μL) were successively added into each chamber every 5 min.

### 4.8. Proteomic Analysis

An integrated approach involving isobaric tags for relative and absolute quantification (iTRAQ) labeling, HPLC fractionation, and mass spectrometry-based quantitative proteomics to quantify changes in the whole proteome of rat brain samples (hippocampus and PFC) was used. Brain samples from six SHAM rats and six OVX rats were taken, and the proteins in the samples were extracted, concentrated, and digested with trypsin respectively. Then, the obtained peptides were labeled with iTRAQ-8plex reagents (Sigma Aldrich, Merck KGaA, Darmstadt, Germany) in accordance with the manufacturer’s protocol. The efficiency of iTRAQ labeling was above 96%. After being labeled, the peptides were fractionated by high-pH reversed-phase HPLC. For high-performance liquid chromatography/tandem mass spectrometry (LC-MS/MS) analysis, the peptides were dissolved in 0.1% formic acid, loaded onto a reversed-phase precolumn (Acclaim PepMap 100; Thermo Fisher Scientific, Waltham, MA, USA), and then separated using a reversed-phase analytical column (Acclaim PepMap RSLC; Thermo Fisher Scientific). The resulting peptides were analyzed through a Q Exactive^TM^ Plus hybrid quadrupole Orbitrap mass spectrometer (Thermo Fisher Scientific, Waltham, MA, USA). To identify and quantify proteins, the resulting MS/MS data were analyzed by MaxQuant with an integrated Andromeda search engine (v.1.4.1.2), and tandem mass spectra were searched against the Uniprot-rat database (32,983 sequences).

As reported proteomic analysis of brain samples [56], we defined observed proteins with iTRAQ ratios of >1.15 or <0.87 coupled with *p* < 0.05 as differentially expressed proteins (DEPs). Benjamini–Hochberg correction was used to control the false discovery rate (FDR), and then DEPs were verified with a threshold of FDR < 0.1. Furthermore, the protein–protein interaction (PPI) network of DEPs was analyzed by The Search Tool for the Retrieval of Interacting Genes (STRING) database (v11.5, http://string-db.org/, accessed on 8 November 2025, STRING Consortium, Zurich, Switzerland) and visualized by Cytoscape Software (v3.10.3, https://cytoscape.org/, accessed on 8 November 2025, Cytoscape Consortium, San Diego, CA, USA).

### 4.9. qRT-PCR Analysis

RNA was isolated from the intestinal tissues using a Trizol-based (Invitrogen, Carlsbad, CA, USA) extraction method. Two ug of total RNA was used for reverse transcription to generate cDNA by PrimeScript RT Reagent Kit with gDNA Eraser (Takara, Kusatsu, Shiga, Japan). qPCR was performed in triplicate using SYBR green, and data were analyzed using the ^ΔΔ^CT method relative to Rpl32. Any samples with amplification values below the limit of detection were assigned a value of 0. The primer sequences used in this study are listed in Table S2.

### 4.10. Statistical Analysis

Sample size calculations and statistical analyses were conducted using SPSS software (Version 26.0; SPSS Inc., Chicago, IL, USA). Normality was tested with the Shapiro–Wilk test, and equal variance was evaluated before analysis of variance analysis. The difference between the two groups was assessed using unpaired Student’s *t*-test (two-tailed) or Mann–Whitney U test. Comparisons among multiple groups was assessed with one-way or two-way analysis of variance (ANOVA), with or without repeated measures, followed by the Tukey, Dunnett, Newman–Keuls, or Bonferroni post hoc test. Correlations were assessed by linear regression; *p* < 0.05 was considered statistically significant.

## 5. Conclusions

In young rats, both EMO and EST prevent OVX-induced cognitive deficits and depressive behaviors, and attenuate the neuronal loss and synaptic abnormalities in brains of OVX rats. Unlike EST, EMO did not increase the blood estrogen level and brain ERα level of OVX rats. EMO elevated intestinal mRNA levels of proGCG and PCSK1, two key molecules involved in GLP-1 production, via ERβ-dependent pathway, thus up-regulated the circulating GLP-1 levels. In the brains of OVX rats, EMO up-regulated the level of GLP-1R, which is implicated in cognition, emotion, and also motor control [18]. The enhanced GLP-1/GLP-1R signaling may be one of the important pathways for EMO to improve the brain functions in estrogen-deficit rats.

## Data Availability

The raw data supporting the conclusions of this article will be made available by the authors, without undue reservation.

## Supplementary Materials

The following supporting information can be downloaded at: https://www.mdpi.com/article/10.3390/ijms27083414/s1.
