# Supplementary material for: Emodin Promoted Intestinal Secretion of GLP-1 and Limited Cognitive Deficits in Young Bilateral Ovariectomized Rats
Source: Int J Mol Sci. 2026 Apr 10;27(8):3414. doi: 10.3390/ijms27083414 (PMC13116777; doi:10.3390/ijms27083414)
Supplement: Supplementary file 1 [file ijms-27-03414-s001.zip › ijms-4198823-supplementary.pdf]

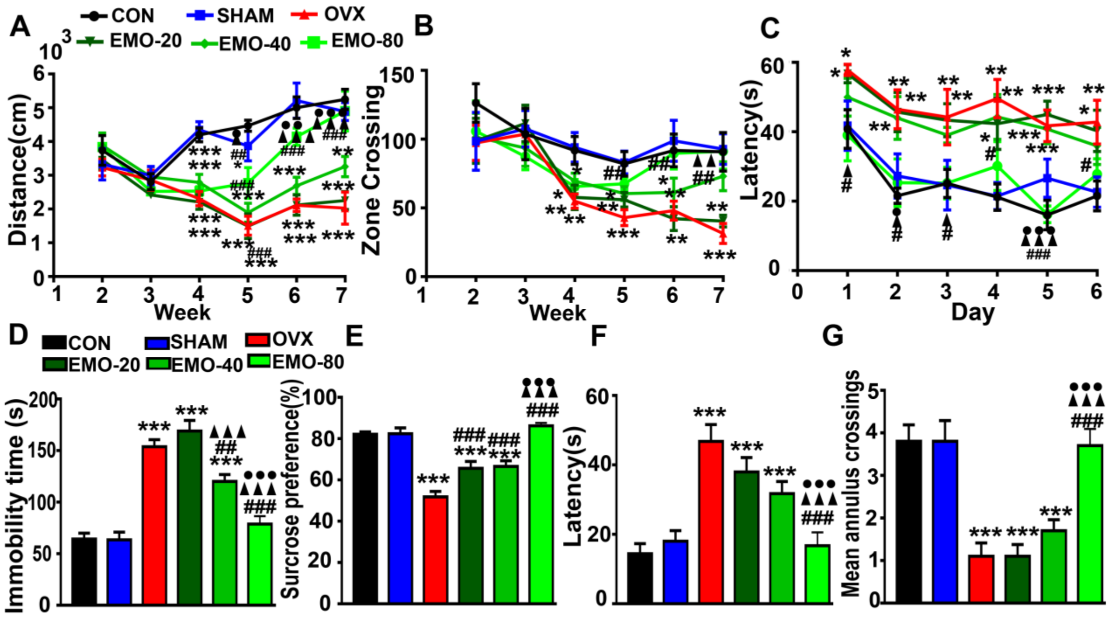


**Supplementary Figure S1. Emodin prevents depressive-like behavior and cognitive deficits in young rats after OVX.** 3-month-old female Sprague-Dawley (SD) rats were divided into control (CON, n=10), sham (SHAM, n=10) and ovariectomized (OVX, n=40) groups. OVX rats were treated with emodin (EMO) two weeks after surgery (20, 40 and 80 mg/kg/day, p. o.; n=10) for six consecutive weeks. Behavioral tests used included SPT, FST, OPT, and MWMT. From weeks 2–7 postoperatively, total distance (**A**) and number of zone crossings (**B**) by OFT of rats was recorded. In week 7, immobility time within 5 minutes in FST (**D**) and the sucrose preference data (**E**) were recorded. In week 8, platform search latency was evaluated on MWMT in the learning phase (**C**), while first-time latency for platform crossing (**F**) and average annulus crossing (**G**) within 1 minute in the memory phase were recorded. Data are presented as mean ± SEM. # $p<0.05$, ## $p<0.01$, ### $p<0.001$, *vs* OVX rats; * $p<0.05$, ** $p<0.01$, *** $p<0.001$, *vs* SHAM rats; ▲ $p<0.05$, ▲▲ $p<0.01$, ▲▲▲ $p<0.001$, *vs* EMO-20 rats; ● $p<0.05$, ●● $p<0.01$, ●●● $p<0.001$, *vs* EMO-40 rats.

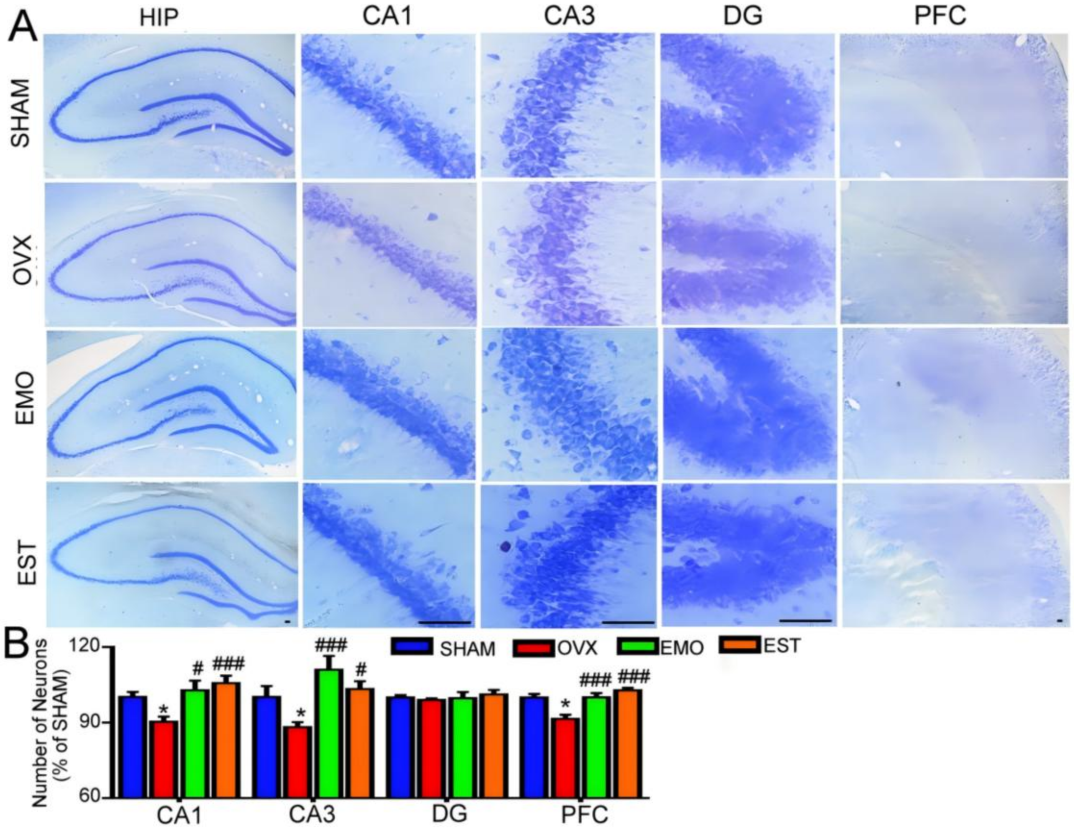


**Supplementary Figure S2. Emodin attenuates neuronal loss in OVX rats.** 8 weeks after surgery, neurons in the hippocampal and PFC regions of SHAM, OVX, EMO and EST group rats were quantified by Nissl staining (**A,** scale bar=200μm; n=3/group) and neuron counting (**B**). Data are presented as mean ± SEM. * $p<0.05$, ** $p<0.01$, *** $p<0.001$, *vs* SHAM rats; # $p<0.05$, ## $p<0.01$, ### $p<0.001$, *vs* OVX rats.

**Table S1. The primary antibodies used in this study, including antibody name, manufacturer, catalog number, and dilution ratio for each experiment.**

| Antibody | Epitopes | mAb/pAb | Dilution | Source |
|---|---|---|---|---|
| ERα | Total ERα | pAb | 1:500 (WB) | Santa Cruz Biotechnology |
| ERβ | Total ERβ | pAb | 1:1000 (WB) | Abcam |
| Ras | Total Ras | pAb | 1:1000 (WB) | Cell Signaling Technology |
| ERK | Total MAPK ERK1/2 | pAb | 1:1000 (WB) | Cell Signaling Technology |
| p-ERK | p-ERK at Thr202/Tyr204 | pAb | 1:1000 (WB) | Cell Signaling Technology |
| STIM2 | Total STIM2 | pAb | 1:1000 (WB) | Prosci |
| TRPC1 | Total TRPC1 | pAb | 1:200 (WB) | Sigma |
| GluR1 | Total GluR1 | pAb | 1:1000 (WB) | Millipore |
| p-GluR1 | p-GluR1 at Ser845 | pAb | 1:1000 (WB) | Thermo Fisher Scientific |
| NR2B | Total NR2B | pAb | 1:1000 (WB) | Cell Signaling Technology |
| p-NR2B | p-NR2B at tyr1472 | pAb | 1:1000 (WB) | Abcam |
| PSD95 | Total PSD95 | pAb | 1:500 (WB) | Cell Signaling Technology |
| Synapsin 1 | Total synapsin 1 | pAb | 1:1000 (WB) | Millipore |
| CaMKIIα | Total CaMKIIα | mAb | 1:1000 (WB) | Thermo Fisher Scientific |
| p-CaMKIIα | p-CaMKIIα at Thr 286 | pAb | 1:1000 (WB) | Santa Cruz Biotechnology |
| proGCG | Total proGCG | mAb | 1:500 (IF) | Abclonal |
| ERβ | Total ERβ | mAb | 1:500 (IF) | Abclonal |
| DM1A | α-tubulin | mAb | 1:1000 (WB) | Cell Signaling Technology |

**Table S2. The primer sequences used for qRT-PCR analysis in this study, including gene name, forward primer sequence, reverse primer sequence, and amplicon length.**

| Gene | Sequence (5' to 3') | Length |
|---|---|---|
| ERα | F: CCCTGCCACTCCACATACAA<br>R: GGATGAGCCACCCTGCTG | 175 |
| ERβ | F: TCGTTCTGGACAGGGATGAG<br>R: TCTGTCACCGCGTTCAGTAG | 226 |
| proGCG | F: GTTCCCAAAGGAGCTCCACC<br>R: GCTGGGAATGATCTGGCGT | 170 |
| PCSK1 | F: CGGTGTCAAACAGGGGAGAC<br>R: CTGCATACCAGGGTGACAGG | 164 |
| GLP-1R | F: CAGGTCTCTTCTGCAACCG<br>R: ATGCCCTTGGAGCACACTAC | 126 |
| GAPDH | F: GAACATCATCCCTGCATCCA<br>R: CCAGTGAGCTTCCCGTTCA | 143 |
